# A missense mutation in *ITGB6* causes pitted hypomineralized amelogenesis imperfecta

**DOI:** 10.1093/hmg/ddt616

**Published:** 2013-12-06

**Authors:** James A. Poulter, Steven J. Brookes, Roger C. Shore, Claire E. L. Smith, Layal Abi Farraj, Jennifer Kirkham, Chris F. Inglehearn, Alan J. Mighell

**Affiliations:** 1 Leeds Institutes of Molecular Medicine, University of Leeds, Leeds LS9 7TF, UK; 2 School of Dentistry, University of Leeds, Leeds LS2 9LU, UK

## Abstract

We identified a family in which pitted hypomineralized amelogenesis imperfecta (AI) with premature enamel failure segregated in an autosomal recessive fashion. Whole-exome sequencing revealed a missense mutation (c.586C>A, p.P196T) in the I-domain of integrin-β6 (ITGB6), which is consistently predicted to be pathogenic by all available programmes and is the only variant that segregates with the disease phenotype. Furthermore, a recent study revealed that mice lacking a functional allele of Itgb6 display a hypomaturation AI phenotype. Phenotypic characterization of affected human teeth in this study showed areas of abnormal prismatic organization, areas of low mineral density and severe abnormal surface pitting in the tooth's coronal portion. We suggest that the pathogenesis of this form of AI may be due to ineffective ligand binding of ITGB6 resulting in either compromised cell–matrix interaction or compromised ITGB6 activation of transforming growth factor-β (TGF-β) impacting indirectly on ameloblast–ameloblast interactions and proteolytic processing of extracellular matrix proteins via MMP20. This study adds to the list of genes mutated in AI and further highlights the importance of cell–matrix interactions during enamel formation.

## INTRODUCTION

Enamel is the hardest human tissue and when formed correctly has the capacity to remain functional in to very old age. Amelogenesis imperfecta (AI) is the collective term for a heterogeneous group of conditions characterized by genetically determined defects in tooth enamel biomineralization which lead to premature clinical failure. Typically, all teeth of the primary and secondary dentitions are affected, with variations in phenotype being influenced by the underlying genotypes (1). The impact of AI on affected individuals, their families and those providing longitudinal care is considerable (2).

Amelogenesis is a stepwise process conserved between species (3), yet the precise mechanisms underlying each phase are not well understood. The basic unit of enamel structure is the prism (or rod), with each prism representing a bundle of nanocrystals of calcium hydroxyapatite mineral (Ca_10_[PO_4_]_6_[OH]_2_) (HAP). The physical properties of mature enamel are a result of its high mineral content and the complex, though ordered, spatial inter-relationship and orientation of the enamel prisms. For correct enamel formation to occur, ameloblasts must undergo four main stages: pre-secretory, secretory, transition and maturation (4). Briefly, ameloblasts grow in length and secrete an enamel matrix at their apical surface which forms the initial layer of aprismatic enamel. As the ameloblasts retreat away from the dentine, they lay down an extracellular matrix within which the hydroxyapatite crystals begin to form. Each enamel prism reflects the migration path of an ameloblast, which is not straight but includes several changes in direction. The highly organized interlocking prismatic pattern resulting from the concerted movement of ameloblast cohorts provides the structure that is key to the physical strength of the final enamel (5). During the maturation stage, ameloblast-mediated proteolytic destruction and removal (via secretion of the protease KLK4) of organic material from the matrix and ameloblast-mediated ion exchange are required for HAP crystals to grow in both thickness and width, until almost the entire tissue volume is occluded by mineral. By the end of the maturation stage, the newly formed enamel contains a mineral content of ∼95% (by weight) (6), but due to the loss of cells from the crown surface on tooth eruption, it is without capacity for cellular repair.

AI can be sub-classified on the basis of the volume of enamel matrix formed and its subsequent mineralization. Prior to tooth eruption, hypomineralized AI has a near-normal enamel matrix volume that is not normally mineralized. Within the spectrum of hypomineralized AI, there are two subtypes that typify the two extremes: hypocalcified and hypomaturation AI. In hypocalcified AI, the enamel may be so soft that it can be scraped away by hand, whereas in hypomaturation AI the enamel is hard yet brittle and prone to fracture off. In contrast, in hypoplastic AI there is failure of enamel matrix formation. In its most extreme form, a very thin layer of enamel, which may be hard or soft, covers the underlying dentine. As such, the tooth crowns have a markedly reduced enamel volume and an abnormal morphology clinically. Focal hypoplasia in the form of pits or grooves may occur within hypomineralized AI reflecting localized areas where enamel matrix formation has been incomplete.

An insight into enamel biomineralization has been gained from the identification of AI-causing mutations in genes encoding enamel matrix proteins (*AMELX*, MIM 30039; *ENAM*, MIM 606585), enamel matrix proteolytic enzymes (*KLK4*, MIM 603767; *MMP20*, MIM 604629), an ion transporter (*SLC24A4*, MIM 609840) and a putative crystal nucleator (*C4orf26*, MIM614829) (7–12). However, the identification of AI-causing mutations in *FAM83H* (MIM 611927) and *WDR72* (MIM 613214), which are of unknown functions, shows that much remains to be understood (13,14).

Mutations in genes with an important role in cell–cell and cell–matrix adhesion, such as *ITGB4* (MIM 147557) and *LAMB3* (MIM 150310), have been identified in patients with isolated and syndromic AI (15–17). The finding that mutations in these genes, encoding integrins and laminins, cause AI indicates that cell–cell and cell–matrix interactions play a vital role in amelogenesis. Recently, an *Integrin-β6* (*Itgb6*) null mouse was described with a hypomaturation AI phenotype (18), but to date no human mutations have been identified in this gene as a cause of AI.

Here, we report findings from a consanguineous family with autosomal recessive pitted hypomineralized AI. We show that a missense mutation of a highly conserved residue in ITGB6 is the cause of the condition in this family, and that the enamel phenotype is similar to that described for the *Itgb6*-null model, except that prism organization is not completely lost in the human case*.* Based upon our detailed phenotyping and recently published data of others, we suggest a potential pathogenic mechanism for AI in these patients based on ITGB6 activation of transforming growth factor-β (TGF-β) and subsequent MMP20 activation via Runx2.

## RESULTS

### Dental phenotype and SNP mapping

**Figure 1.**
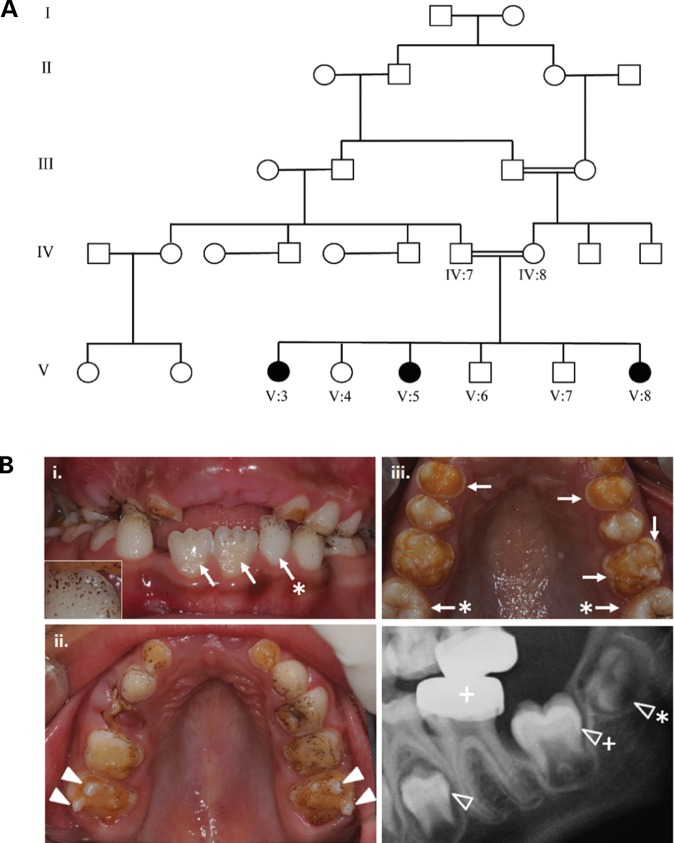
Family pedigree and clinical dental phenotypes for AI-23. (**A**) Pedigree of family AI-23 recording the three affected individuals within a consanguineous family. DNA was available for all labelled individuals. (**B**) (i) and (ii) The clinical appearances for V8 aged 7 years of the early mixed dentition with premature enamel failure. Surface enamel pitting is evident on many teeth, including the partially erupted permanent lower incisors (arrows) and via the ‘speckled black’ appearances due to exogenous staining in the pits. Inset image is a higher magnification image of the deciduous tooth (arrow *) highlighting the pitting. The retention of enamel over the cusps of the permanent molar teeth (arrow heads) at this stage highlights the dramatic loss of enamel from the rest of the dental crown, even though these teeth have been in the mouth for a short period of time. (iii) Clinical appearances of the upper dentition for V3 aged 9 years illustrating how the enamel can fracture cleanly away leaving a shoulder of remaining enamel at the cervical margin (arrows). The enamel of the newly erupted second permanent molar teeth has yet to fail (arrow *). (iv) Dental radiograph of V3 aged 7 years confirming a near-normal enamel volume in the unerupted second premolar (arrowhead) and second molar (arrowhead +) lower permanent teeth with a clear difference in radiodensity between enamel and dentine, consistent with what is observed in normal teeth. The first lower permanent molar tooth has been restored with a metal crown (+). The crown of the permanent lower third molar tooth is starting to mineralize (arrowhead *).

We identified a consanguineous family (AI-23) living locally, but originating from Pakistan, in which pitted hypomineralized AI segregates with an autosomal recessive mode of inheritance in the absence of any other co-segregating diseases (Fig. 1). Affected individuals presented with poor dental aesthetics and associated pain, for example on eating or drinking.

To identify the genetic basis of AI in this family, individuals V:3 and V:5 were genotyped using Affymetrix 6.0 SNP microarrays, and shared regions of homozygosity were identified using AutoSNPa (19). Six regions of homozygosity spanning ∼73 Mb were identified (Table 1), none of which overlapped with previously published AI loci. We therefore decided to use whole-exome sequencing to identify the cause of disease in this family.

**Table 1.** Summary of variants in AI-23 candidate disease regions and variants discovered by exome sequencing

Region	Size (Mb)	Variants not in dbSNP 129 or MAF ≤ 1%	… and predicted functional	… and segregates with the disease
Chr2:154,600,940–173,240,770	18.64	31	3	1
Chr7:154,812,233–157,683,557	2.87	4	0	–
Chr10:80,853,408–90,425,474	9.57	8	0	–
Chr11:100,244,686–113,840,625	13.60	21	1	0
Chr12:4,023,387–21,726,294	17.70	41	0	–
Chr22:22,798,234–33,658,203	10.86	33	0	–
Total	72.6	138	4	1

The total variants identified in each region are shown.

### Whole-exome and Sanger sequencing

Genomic DNA from individual V:3 was subjected to whole-exome sequencing using a 100 bp paired-end protocol on an Illumina Hi-Seq 2000 sequencer. Sequence reads obtained were aligned to the human reference sequence (GRCh37) using Bowtie2 software. The resulting alignment was processed in the SAM/BAM format using the SAMtools, Picard and GATK programs in order to correct alignments around indel sites and mark potential PCR duplicates. Following post-processing and duplicate removal, a mean depth of 64 reads was achieved for targeted exons in the homozygous regions, with 98.2% of these bases covered by at least five reads.

Indel and single-nucleotide variants within the six candidate regions were called in the VCF format using the Unified Genotyper function of the GATK program, revealing a total of 1847 variants. Using the dbSNP database at NCBI (http://www.ncbi.nlm.nih.gov/projects/SNP/), any variants present in dbSNP 129, together with those variants present in dbSNP 137 with a minor allele frequency (MAF) ≥1%, were then excluded. The 138 remaining variants were annotated using the SeattleSeq Variation Annotation server v.137 (http://snp.gs.washington.edu/SeattleSeqAnnotation137/), which identified 134 as either synonymous or deep intronic variants. Of the remaining four variants, two are present in dbSNP with an MAF of <1.0%. These are rs147066399 (NM_213621:c.736C>T [p.R246W] in *HTR3A* (MIM 182139)) with an MAF of 1/4545; and rs113262393 (NM_033394.2:c.1413T>G [p.S471R] in *TANC1* (MIM 611397)) with an MAF of 9/2190. In addition, rs147066399 in *HTR3A* has been observed once in 87 South Asian samples in an in-house exome dataset. A third missense variant, p.W879S in *LRP2* (NM_004525:c.2636C>G (MIM 600073)), has also been observed in heterozygous form twice in 87 South Asian samples in our in-house exome dataset. Furthermore, these three variants do not segregate fully with the disease phenotype in the AI-23 family.

**Figure 2.**
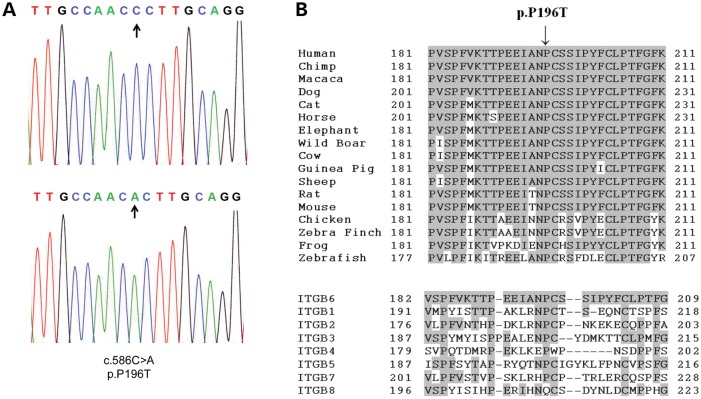
Electropherogram of mutation and conservation of the P196T variant. (**A**) Representative electropherogram of the ITGB6 mutation in an affected member of family AI-23 alongside the wild-type sequence. Arrows indicate the localization of the variant. (**B**) Conservation of the P196 residue in orthologues (upper) and paralogues (lower). Conserved residues are highlighted. Human (NP_000879), Chimp (XP_001149234), Macaca (XP_001094740), Dog (XP_857148), Cat (XP_003990848), Horse (XP_001492914), African Elephant (XP_003406050), Wild Boar (NP_001090892), Cow (NP_777123), Guinea Pig (XP_003478725), Sheep (NP_001107244), Rat (NP_001004263), Mouse (NP_067334), Chicken (XP_422037), Zebra Finch (XP_002193421), Frog (NP_001090775), Zebrafish (XP_003199474), Human ITGB1 (NP_596867), ITGB2 (NP_001120963), ITGB3 (NP_000203), ITGB4 (NP_000204), ITGB5 (NP_002204), ITGB7 (NP_000880) and ITGB8 (NP_002205).

The remaining variant, p.P196T in *ITGB6* (NM_000888:c.586C > A) (Fig. 2A), was not present in dbSNP 137 nor in the in-house exome dataset. The c.586C>A variant in *ITGB6* was further excluded in a panel of 174 control chromosomes from an ethnic diversity panel and was found to segregate perfectly with the disease phenotype in family AI-23. Both parents were heterozygous for the change. Investigation of the P196 residue, present within the I-domain of ITGB6, revealed it to be fully conserved in all orthologues and paralogues, with only ITGB8 differing at this residue (Fig. 2B), suggesting that it plays a crucial role in the function of the protein. Furthermore, the bioinformatic prediction packages PolyPhen2, MutationTaster, Sift, Blosum62, Provean and MutPred consistently predicted this to be a likely pathogenic change (Supplementary Material, Table S1).

In light of these findings, we investigated a panel of 44 unrelated individuals diagnosed with hypomineralized AI from diverse backgrounds. PCR amplification and Sanger sequencing of all the coding exons and intron–exon boundaries of *ITGB6* were therefore performed. No further pathogenic variants were identified.

### Enamel phenotyping

**Figure 3.**
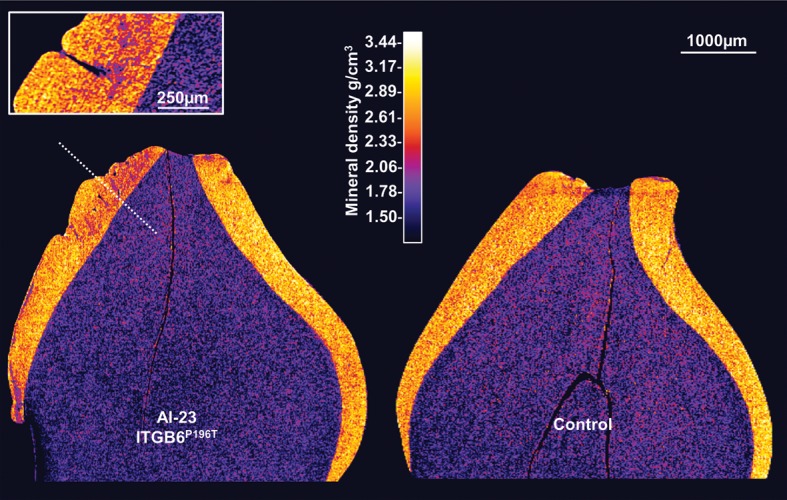
Phenotypic analyses of enamel: µCT. (**A**) µCT confirmed a reasonable enamel volume in affected teeth, but with a multiple enamel surface and sub-surface abnormalities. Particularly striking were the regions of enamel exhibiting reduced mineral density and pits running for the enamel surface deep in to the bulk of the enamel.

To gain further insights into why the affected enamel undergoes premature clinical failure, we undertook laboratory investigations of the remaining enamel on affected deciduous teeth and matched normal teeth. Figure 3 shows representative micro-computed tomography (µCT) buccal–lingual sections through an affected deciduous canine tooth and a matched control. The scans have been calibrated for mineral density and mapped in colour. Both teeth exhibit wear at the cusp tips where the outer covering of mineral-dense enamel has worn away to expose the less-mineralized underlying dentine. However, the affected tooth was characterized by an abnormally roughened lingual surface with areas of sub-surface enamel exhibiting reduced mineral density, though in general, affected enamel was comparable in thickness with control enamel, suggesting that the enamel matrix volume was not markedly affected by the mutation. The apparent sub-surface voids or ‘holes’ in the affected enamel shown in Figure 3 are actually pits running orthogonally to the section. An orthogonal section (inset) along the plane indicated by the white dotted line reveals a typical pit running from the enamel surface towards the enamel–dentine junction. By comparison, the control tooth exhibits enamel of a more uniform mineral density comparable with previous reports of deciduous molar enamel density which ranged from 2.69 to 2.92 g/cm^3^ (20) and a smooth enamel surface.

**Figure 4.**
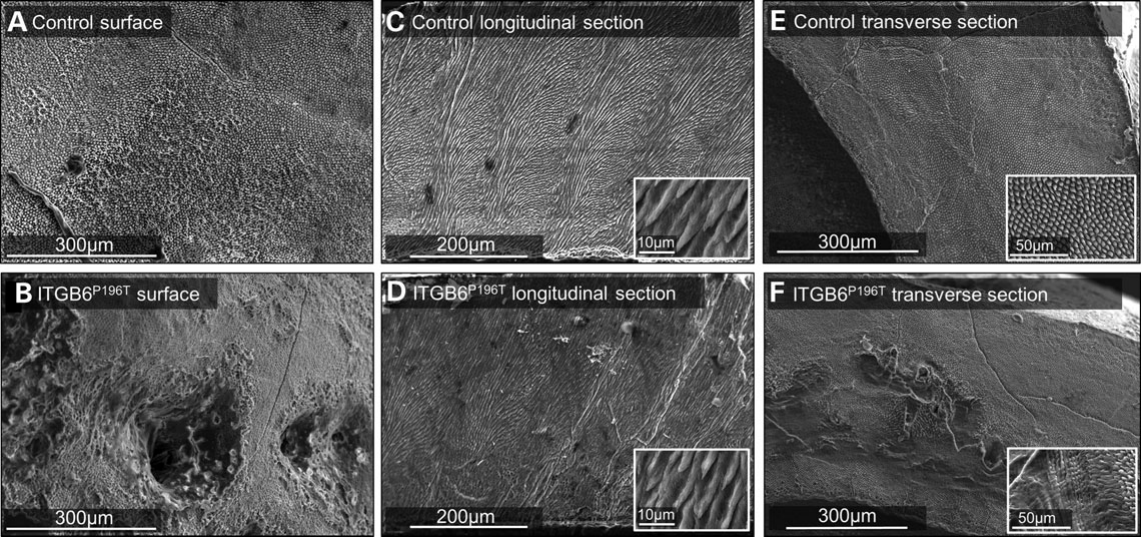
Phenotypic analyses of enamel: SEM. (**A**) SEM of the etched control enamel surface showed the characteristic appearance of arrays of enamel prisms terminating at the surface. (**B**) In contrast, the affected enamel was punctured by numerous pits and the prism array was more obscure. (**C**) SEM of the internal prim architecture of control enamel in longitudinal section revealed the characteristic sinusoidal pattern of prism cohorts giving rise to Hunter–Schreger bands. (**D**) The prism architecture in affected enamel was less regular and clear Hunter–Schreger bands were less distinct. The inset images in (C) and (D) show that the individual prism structure at the micron level in control and affected enamel is indistinguishable by SEM in some areas. (**E**) SEM of transversely cut sections through control cuspal enamel showed the characteristic array of prisms themselves in the transverse section (higher magnification inset). (**F**) In affected enamel, the inner enamel layer is structurally abnormal with loss of prism organization. Structural features designed to provide mechanical stability that depend on the correct inter-relationship between prism cohorts (e.g. Hunter–Schreger bands) will be compromised in this enamel (inset shows higher magnification details).

At the ultrastructural level and following surface etching (Fig. 4A), scanning electron microscopy (SEM) revealed an abnormal surface in affected teeth associated with the pits previously identified using µCT (Fig. 4B). In control enamel (Fig. 4C), SEM of the internal enamel structure in teeth cut longitudinally along the buccal–lingual midline revealed a typical enamel architecture comprising cohorts of enamel prisms changing direction relative to each other (reflecting the movement of ameloblast cohorts) to generate Hunter–Schreger banding (21). In contrast, our data suggest that prism orientation in the affected enamel may be disturbed and the synchronous changes in direction of the enamel prisms responsible for generating the Hunter–Schreger bands are compromised (Fig. 4D). At the microscale, the structure of individual prisms in both control and affected enamel is indistinguishable using SEM (inset Fig. 4C and D). SEM of internal enamel structure revealed by cutting transversely through the cuspal region shows prisms in control teeth evenly arrayed across the cut surface (Fig. 4E). In contrast, affected enamel exhibits areas of a grossly disorganized prism architecture in the inner third of the enamel (Fig. 4D).

## DISCUSSION

We identified a family of Pakistani origin segregating pitted hypomineralized AI in an autosomal recessive manner. SNP chip analysis and whole-exome sequencing revealed a homozygous ITGB6 mutation resulting in the missense, p.P196T change as the only plausible cause. A screen of 44 further unrelated families revealed no further mutations, indicating that this is a rare cause of AI in the cohort studied. Disruption of ITGB6 function has been linked with aged-related chronic obstructive pulmonary disease (COPD) (22). The affected individuals in the family studied are young, and it is too early to know whether the missense change identified will predispose them to COPD.

The integrins are a major family of cell surface-adhesion receptors involved in cell–cell, cell–matrix and cell–pathogen interactions (23). Each integrin is composed of α and β subunits, which are non-covalently bound together with some promiscuity in subunit partnerships. ITGB6 commonly binds integrin-αv, forming integrin-αvβ6, an epithelial cell-specific integrin, which is a predominant binder to the arginine–glycine–aspartic acid (RGD) amino acid motif (24,25). Others have described the binding of cell surface expressed integrin-αvβ6 to RGD motifs which are widely distributed, for example in extracellular matrix proteins such as fibronectin, vitronectin and tenascin-C as well as the latency-associated peptide (LAP) of TGF-β1 and TGF-β3. The missense variant identified in this study is present within the β6-integrin I-domain, which is involved in α-integrin, divalent cation and ligand binding. Furthermore, the mutated 196P residue is in a highly conserved region next to one of the conserved extracellular cysteine residues important for integrin structure and function (Fig. 2B).

Matrix–cell binding proteins such as fibronectin are important in the epithelial–mesenchymal interactions during the pre-secretory-stage amelogenesis (26), but are absent during the later stages (27). However, the patients described herein have teeth with a near-normal enamel volume, but with structurally compromised enamel, consistent with a defect that occurred after the pre-secretory phase of amelogenesis. This suggests that problems due to integrin–fibronectin RGD binding may not be the primary causal effect in the case of this mutation.

The AI phenotype in this family shares some similarity to that of mice lacking a functional allele of *Itgb6*, with both falling within the hypomineralized AI spectrum. The cause of hypomineralization in affected teeth could be a reduction in prism density *per se* or prism density may be normal but the individual enamel crystallites within each prism may have failed to grow to their normal maximum dimensions. The laboratory phenotyping of affected human enamel presented here provides insight into why the enamel is prone to premature failure post-eruption. Although enamel of normal thickness is produced during amelogenesis of affected individuals and the normal elements of enamel architecture; i.e. enamel prisms are present, µCT and SEM indicate that the enamel is compromised by defects running from abnormal enamel surface defects (pits) towards the enamel–dentine junction. In addition, the affected teeth appear to contain localized regions of hypomineralization; especially in the cuspal regions. There is also indication that the macro-organization of enamel prisms in affected teeth is abnormal as evidenced by the SEM data which would be expected to impact the mechanical properties of the enamel that depend on the organization of prism cohorts. In normal enamel development, the synchronous movement of cohorts of ameloblasts gives rise to equivalent cohorts of prisms that follow a sinusoidal track from the enamel–dentine junction to the enamel surface. This gives rise to the Hunter–Schreger bands which serve to inhibit crack propagation in the enamel. Based on our preliminary phenotyping, we suggest that affected enamel is structurally compromised by pitting, localized hypomineralization and disturbed prism architecture, all of which undermine the ability of the enamel to resist the stresses generated during mastication. In summary, the enamel in affected members of this family is consistent with hypomineralized AI with focal hypoplasia in the form of pits.

ITGB6 binding to LAP of TGF-β1 activates the cytokine (28). TGF-β1and its associated receptor are expressed by secretory-stage ameloblasts (autocrine signalling) and in ameloblast cell lines, TGF-β1 promotes the expression of MMP20 (29) via Runx2 (30). MMP20 is a crucial enzyme responsible for the processing of the developing enamel extracellular matrix. Failure to correctly process the enamel matrix proteins (e.g. amelogenin) would lead to retention of matrix proteins in maturing enamel and prevent the enamel mineralizing fully. Retained amelogenin has been identified in other AI isoforms (31).

MMP20 has also been implicated in controlling ameloblast–ameloblast cell contact by cleaving an extracellular domain of cadherin, which in turn controls the ability of ameloblasts to execute their movements relative to each other. These movements are essential to generate the correct prism architecture (32,33) needed to produce functional, mature enamel. In MMP20 null mice, normal prism decussation is abolished, matrix proteins are retained and erupting enamel undergoes rapid failure (34). Thus, there is a putative molecular trail linking mutated ITGB6 to prism disorganization and the eruption of hypomineralized enamel (caused by retention of enamel matrix proteins) due to compromised MMP20 expression by epithelia-derived ameloblasts. That Itgb6 is able to activate TGF-β1 in oral epithelia tissue is supported by the Itgb6-mediated activation of TGF-β1 in gingival epithelium (35). However, in argument against the hypothesis presented above, TGF-β1 activation in mouse enamel organ, as determined by Smad1/2 phosphorylation, and MMP20 expression was similar in WT and Itgb6 knockout animals (18). It is unclear what species–species differences exist between human and mouse amelogenesis in terms of TGF-β1 signalling, and it is equally unclear how expression of ITGB6 with a missense change compares with a complete ITGB6 knockout; certainly, the mouse Itgb6 knockout phenotype is different from the human example presented here as at least some degree of prism organization is retained in affected human enamel. Clearly, more work is required to support a mechanism by which ITGB6 causes AI via a TGF-β1 induced MMP20 activity, but we present the hypothesis as a frame work on which to base future studies.

The distribution of enamel defects in AI resulting from a mutation in ITGB6 was not even throughout the anatomical crown. Pitting and areas of hypomineralization were localized to the coronal portion of the tooth, the thinner cervical enamel being spared and of normal mineral density and appearance. Ameloblasts travel a shorter distance in elaborating cervical tissue and their activity takes place over a shorter timescale (as apposition rates are similar irrespective of the location on the tooth) (36). Mathematical modelling has shown that the strains on ameloblasts producing enamel in the coronal region of the tooth are much greater than those experienced by their cervical counterparts, as the coronal portion of the tooth expands greatly during amelogenesis yet the numbers of ameloblasts do not increase (37). In simple terms, as coronal enamel is laid down, the surface area of the enamel increases and the monolayer sheet of ameloblasts covering the increasing surface must be able to modulate cell-to-cell contact to prevent stress-related holes being formed in what is normally a continuous cell monolayer. Any compromise in the ability of cells to modulate their contacts with adjacent cells would leave the ameloblast monolayer more susceptible to stress-induced disruption. Any holes appearing in the ameloblast monolayer during enamel secretion would be recorded in the enamel as a pit; similar to the pitting found in affected enamel in this study.

**Figure 5.**
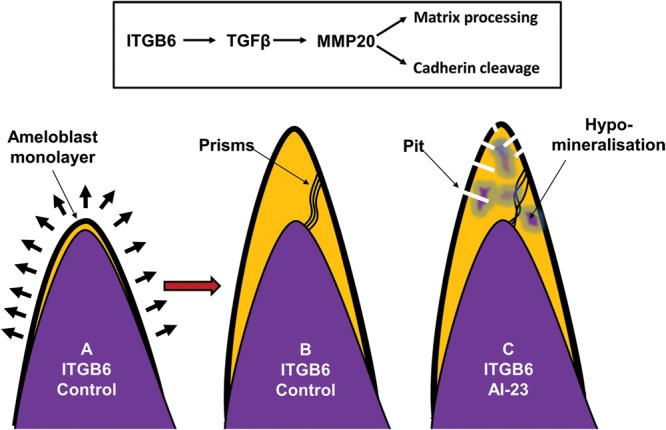
Cartoon summarizing the hypothesis presented here to explain the mechanism underpinning the AI subtype described. (**A**) At the beginning of normal enamel secretion, an army of ameloblasts, present as a monolayer, migrates away from the preformed dentine surface leaving the enamel matix in their wake. (**B**) As the cuspal enamel volume increases the ameloblasts modulate cell–cell contacts to cope with the stresses encountered by the monolayer being required to cover an ever expanding area. We hypothesize that ITGB6 upregulates MMP20 expression (via TGFβ activation). This in turn cleaves cadherin, thus allowing ameloblasts to modulate cell–cell contacts to cope with the increasing stress of an expanding enamel surface and to allow cohorts of ameloblast to move relative to one another to produce a sinusoidal prism architecture. MMP20 also processes enamel matrix proteins, which is required for their final degradation prior to the completion of mineralization. In affected enamel, we hypothesize that cadherin cleavage and matrix processing are compromised due to the ITGB6 mutation resulting in breaks in the ameloblast monolayer in the cuspal regions leading to pitting, abnormal prism architecture and retained matrix proteins that inhibit final enamel mineralization.

In summary, if we combine all of the information available to us from this study and the work of others, we are able to suggest a hypothesis for the pathogenesis of AI in these patients (summarized in Fig. 5).

1. The mutation in ITGB6 would be predicted to inhibit activation of TGFβ due to its failure to bind to LAP.
2. This, in turn, would inhibit expression of MMP20 which is essential for matrix processing to permit secondary crystal growth to occur. Failure to process matrix would lead to hypomineralization as observed in our study and in the *Itgb6*-null mouse.
3. MMP20 is also necessary for correct ameloblast–ameloblast cell contact due to its role in cadherin processing. Failure in cell–cell contacts will compromise the ability of ameloblasts to move relative to one another, leading to the abnormal prism decussation seen here (and in the *Itgb6*-null mouse), including the disruption of Hunter–Schreger banding.
4. Mathematical modelling predicts that coronally positioned ameloblasts are under greater strain and thus, the effects of any compromised cell–cell contact would be more severely manifested at the upper part of the tooth compared with the cervical margins. This corresponds to the spatial distribution of pitting that we see in the affected teeth in this study.

We propose therefore that the observed phenotype of the *ITGB6* mutation is due to the combined effects of a lack of MMP20 and compromised ameloblast cell–cell binding resulting in hypomineralization, structural abnormalities and where ameloblast strain is maximal, pitting due to the integrity of the ameloblast monolayer being compromised. Taken together, these developmental defects would result in the pitted hypomineralized AI and failure of enamel function as seen in these patients.

## MATERIALS AND METHODS

### Patients

Members of a family (AI-23), originating from Pakistan, were recruited following informed consent in accordance with the principles outlined in the declaration of Helsinki, with local ethical approval. DNA samples were obtained from family members using either Oragene^®^ DNA sample collections kits (DNA Genotek, ONT, Canada) or via venous blood samples using conventional techniques.

### SNP analysis

Genomic DNAs from two affected individuals were genotyped using Affymetrix 6.0 SNP microarrays by AROS Applied Biotechnology (Aarhus, Denmark). The resulting CEL files were annotated and analysed using autoSNPa to identify shared regions of homozygosity between both the affected individuals (19).

### Exome sequencing

Whole-exome sequencing was performed on genomic DNA using the Nextera Exome Enrichment system (Illumina, CA, USA). In brief, 50 ng of genomic DNA was tagged and fragmented using the Illumina Tagmentation system. Following a clean-up step, tagged DNA was PCR amplified and the subsequent library validated. Validated samples were pooled six per lane with sequencing performed on an Illumina Hi-Seq 2000 using a 100 bp paired-end protocol. Data analysis was performed using Bowtie2, SAMtools, Picard and the Genome Analysis Toolkit (GATK) (38–41).

### PCR and sequencing

Segregation of variants identified by whole-exome sequencing and screening of additional AI families was performed by PCR and Sanger sequencing using standard protocols. Primers to amplify the exons and exon–intron boundaries of *ITGB6* were designed using ExonPrimer (http://ihg.gsf.de/ihg/ExonPrimer.html) and are found in Supplementary Table 2.

### Tooth ultrastructure analysis: X-ray µCT and SEM

An extracted, dried AI-23 deciduous canine was compared with an extracted, dried control deciduous canine using µCT and SEM. The control tooth was obtained with patient consent and ethical approval from a registered tissue bank operated by the School of Dentistry University of Leeds. Teeth were scanned in air together with a calibration phantom comprising a segment of mouse incisor enamel and dentine of known mineral density using a Skyscan 1172 high-resolution X-ray CT scanner (Bruker, Kontich, Belgium) operated at 55 kV; 10watts power with an image pixel size of 2–2.2 µm. The teeth were rotated through 180° and shadow X-ray images captured at 0.27° intervals. The X-rays were filtered through a 0.5 mm Al/Cu filter to reduce beam hardening effects. The shadow images were reconstructed using Skyscan Recon software to generate an image stack comprising sections through the teeth. The Recon software was also used to further correct ring artefacts and beam hardening. The sectional images were further analysed using ImageJ software (National Institutes of Health, Bethesda, MD, USA) and calibrated with respect to mineral density using the mouse incisor as a calibrator.

For SEM, teeth were cut along the buccal–lingual midline or transversely through the cusp region with a diamond cutting disk. The cut surfaces were lightly polished using 2000 grade carborundum paper and etched for 20 s by gentle agitation in 30% phosphoric acid to remove any smear layer. Samples were rinsed in copious amounts of distilled water and dried under vacuum overnight prior to sputter coating with gold. SEM images were obtained using a Hitachi S-3400 operating in secondary electron emission mode at an accelerating voltage of 20 kV and an emission current of 22.1 µA.

## SUPPLEMENTARY MATERIAL

Supplementary Material is available at *HMG* online.

## FUNDING

The project was funded by the Wellcome Trust (grant no. 093113). J.K. is supported by the NIHR Leeds Musculoskeletal Biomedical Research Unit. Funding to pay the Open Access publication charges for this article was provided by the Wellcome Trust.

## Supplementary Material

Supplementary Data
