# Supplementary material for: A missense mutation in ITGB6 causes pitted hypomineralized amelogenesis imperfecta
Source: Hum Mol Genet. 2013 Dec 6;23(8):2189–97. doi: 10.1093/hmg/ddt616 (PMC3959822; doi:10.1093/hmg/ddt616)
Supplement: Supplementary Data [file supp_23_8_2189__index.html]

A missense mutation in ITGB6 causes pitted hypomineralised amelogenesis imperfecta — A missense mutation in ITGB6 causes pitted hypomineralized amelogenesis imperfecta — A missense mutation in ITGB6 causes pitted hypomineralized amelogenesis imperfecta — Supplementary Data 

# A missense mutation in *ITGB6* causes pitted hypomineralized amelogenesis imperfecta

## Supplementary Data

Supplementary Data

**Files in this Data Supplement:**

- Supplementary Data - Doc file
