# Supplementary material for: A missense mutation in ITGB6 causes pitted hypomineralized amelogenesis imperfecta
Source: Hum Mol Genet. 2013 Dec 6;23(8):2189–97. doi: 10.1093/hmg/ddt616 (PMC3959822; doi:10.1093/hmg/ddt616)
Supplement: Supplementary Data [file supp_ddt616_ddt616supp.doc]

**A missense mutation in *ITGB6* causes pitted hypomineralised amelogenesis imperfecta**

***Supplementary Data***

**Supplementary Table 1** - Summary of bioinformatics analyses undertaken to predict the pathogenic nature of the p.P196T missense mutation. URLs: PolyPhen2, <http://genetics.bwh.harvard.edu/pph2/> (1); Mutationtaster, <http://www.mutationtaster.org/> (2); SIFT, [http://**sift**.jcvi.org/](http://sift.jcvi.org/) (3); Blosum62 (4); PROVEAN, <http://provean.jcvi.org/> (5); MutPred, <http://mutpred.mutdb.org/> (6).

‎

| **Mutation** | **PolyPhen2** | **MutationTaster** | **SIFT** | **Blosum62*** | **PROVEAN** | **MutPred** |
| --- | --- | --- | --- | --- | --- | --- |
| p.P196T | Score = 0.988  Probably Damaging | Score = 0.999  Disease Causing | Score = 0  Damaging | -1 | Score = -7.682  Deleterious | Score = 0.855  Loss of catalytic residue |

*Blosum62 scores range from +3 to -4 and negative scores are more likely to be damaging substitutions.

**Supplementary Table 2** – Primers designed using ExonPrimer to amplify the exons and surrounding intronic sequence of *ITGB6*.

| **Exon** | **F Primer (5’-3’)** | **R Primer (5’-3’)** |
| --- | --- | --- |
| 1 | TCAGCGTTGGTCTTGTAACG | TCACAAAAGAGCAATGGAACA |
| 2 | GAGCCATCAGTCTCATAAATGG | TCACTATCAGAAATATCACTGAGGAA |
| 3 | GCAGAAAAACTTTCAATTGCTG | ACAAGACACATTAGCAAGGTAGC |
| 4 | GCGAGTGGCCAGTAGCAA | CTGGCAAGTGCAGCTCAG |
| 5 | GCAGAGTCAGGCCCTTTAGA | AAGGACAGTCCCCATTTCAA |
| 6 | TCCAAGCATAATTGATCCACA | CTGGGGCAACTTGTTAATCC |
| 7 | TGAGCATTGAAATGCCAAAC | CTAATGGCCTTCCCATGTGT |
| 8 | CAGGCTCAAACCCACACTTT | AACATTTAACTGCCTAAATCACATC |
| 9 | TGAACATGATTCACGGTTTCT | GAAAGAAATCCAGCTTCAGTGAG |
| 10-1 | TTTCCGTGAAGCCTTGTTTC | ACACCCCACACTGGAAAGAG |
| 10-2 | ACTGCGAGAGAAGAAGCAGG | AAACTGGAAGGTGCAGAGGA |
| 11 | AAGAGAAAAGATGCCCCTCA | TCTGAGTTTACAAAATGCCACTG |
| 12 | CAGTAAAGCAATATTCAAGCTGC | CGATTCACAGAGGGTCAAGA |
| 13 | TGGAACGTGAGTGAAAACGA | CATGCTACCCAGAGCGATTT |
| 14 | TTTAAGCAAGGCGAAAATGG | AGAAGAAAGCTGAGCCCCTC |
| 15 | CCTCTTCCAATTTGCACTACG | CCAACCTCATTTTGAAGCAA |

**Supplementary References**

1 Adzhubei, I.A., Schmidt, S., Peshkin, L., Ramensky, V.E., Gerasimova, A., Bork, P., Kondrashov, A.S. and Sunyaev, S.R. (2010) A method and server for predicting damaging missense mutations. *Nat. Methods*, **7**, 248-249.

2 Schwarz, J.M., Rodelsperger, C., Schuelke, M. and Seelow, D. (2010) MutationTaster evaluates disease-causing potential of sequence alterations. *Nat. Methods*, **7**, 575-576.

3 Ng, P.C. and Henikoff, S. (2003) SIFT: Predicting amino acid changes that affect protein function. *Nucleic Acids Res.*, **31**, 3812-3814.

4 Henikoff, S. and Henikoff, J.G. (1993) Performance evaluation of amino acid substitution matrices. *Proteins*, **17**, 49-61.

5 Choi, Y., Sims, G.E., Murphy, S., Miller, J.R. and Chan, A.P. (2012) Predicting the functional effect of amino acid substitutions and indels. *PLoS One*, **7**, e46688.

6 Li, B., Krishnan, V.G., Mort, M.E., Xin, F., Kamati, K.K., Cooper, D.N., Mooney, S.D. and Radivojac, P. (2009) Automated inference of molecular mechanisms of disease from amino acid substitutions. *Bioinformatics*, **25**, 2744-2750.
